# High Frequency Ultrasound-Based Evaluation of Clinical Effect of Ephedra-Forsythia-Red Bean Decoction Addition and Subtraction Treatment of Psoriasis Vulgaris

**DOI:** 10.1155/2022/9484230

**Published:** 2022-03-30

**Authors:** Yue Wang, Hongju Lu, Zhongzhu Hu

**Affiliations:** ^1^Department of Dermatology, Wuhan First Hospital, Wuhan 430022, Hubei, China; ^2^Department of Dermatology, Hankou Hospital, Wuhan 430012, Hubei, China; ^3^Department of Dermatology, Huanggang Central Hospital of Yangtze University, Huanggang 438000, Hubei, China

## Abstract

This study aimed to investigate the therapeutic effect of ephedra-forsythia-red bean decoction (a formula of traditional Chinese medicine) addition and subtraction treatment of psoriasis vulgaris based on 22 MHz high-frequency ultrasound, so as to provide reference for the selection of traditional Chinese medicine formulas for psoriasis and the clinical application of ultrasound. 80 patients with psoriasis vulgaris with exterior closing and internal depression syndrome diagnosed and treated in the hospital were divided into an observation group (40 cases) and a control group. Patients in the observation group were received ephedra-forsythia-red bean decoction addition and subtraction; and those in the control group were received traditional Chinese medicine of observation group subtraction of raw ephedra, cinnamomum cassia, addition of fineleaf schizonepeta herb, divaricate saposhnikovia root. 22 MHz high-frequency ultrasonography was also performed. The psoriasis area and severity index (PASI) score and efficacy indicators were compared between the two groups. The results showed that the detection rate of nail malnutrition and psoriasis infiltration in psoriasis by high-frequency ultrasound was significantly higher than that by arthroscopy, and the difference was significant (*P* < 0.05). The total PASI score of the two groups after treatment was significantly lower than that before treatment, and the total PASI score of the observation group was lower than that of the control group, and the difference was statistically significant (*P* < 0.05). The total effective rate of the observation group (87.5%) was significantly higher than that of the control group (67.5%), and the difference was statistically significant (*P* < 0.05). It was found that high-frequency ultrasound can effectively display the condition and prognosis of patients with psoriasis. Ephedra-forsythia-red bean decoction was an effective traditional Chinese medicine formula for the treatment of psoriasis vulgaris.

## 1. Introduction

Psoriasis vulgaris is a common chronic relapsing inflammatory skin disease with characteristic skin lesions [1]. It begins as an inflammatory red papule, ranging in size from milia to mung bean, and then gradually expands or coalesces into brownish-red plaques with well-defined borders; in addition, it is surrounded by an inflammatory blush, marked basal infiltration, and a surface covered with multiple layers of dry grayish or silvery scales [2, 3]. White scales, shiny films, and petechial hemorrhages are important features for the diagnosis of psoriasis. The course of psoriasis vulgaris is slow, and some develop since childhood, lasting for more than ten years or decades, or even protracted for life [4–6]. It is prone to recurrent attacks, and there are also a few who are cured without recurrence. Most patients have aggravated or recurrent symptoms by winter and reduced or disappeared by spring and summer [7]. Generally, mild psoriasis vulgaris generally only requires topical drug therapy, which can control local skin lesions and reduce the systemic dosage and the occurrence of adverse reactions when used in combination with internal drugs [8]. The moderate and severe psoriasis often requires treatment with combination therapy. At present, retinoids are commonly used, for example, acitretin has a shorter half-life in the body than etretinate and a reduced accumulation in the body, thereby reducing side effects, which is a better drug for the treatment of psoriasis, especially moderate and severe psoriasis [9]. In addition to active treatment, daily care is also important, such as not drinking alcohol, eating fewer irritating foods, avoiding physical, chemical substances and drug stimulation.

Ephedra-forsythia-red bean decoction is from Zhang Zhongjing's *Treatise on Febrile Diseases, Diagnosis of Yangming Disease and Pulse Syndrome and Treatment*. It can resolve external pathogens, clear away heat, remove dampness and reduce jaundice, and mainly treat damp-heat jaundice syndrome with external pathogens [10]. The prescription was defined as follows: 6 grams of ephedra, 6 grams of forsythia root, 6 grams of almonds, 10 grams of Chixiaodou, 12 jujubes (smashed), 10 grams of raw white skin (cut), 6 grams of ginger, and 6 grams of licorice (baked). Among them, ephedra, almonds, and ginger were pungent to disperse pathogens, and depressed and sent out stagnant heat; Forsythia Root, Shengzi Baipi, Chixiaodou could clear dampness and heat; and jujube and licorice could harmonize the spleen and stomach. Clinical studies have found that ephedra-forsythia-red bean decoction is a classic and high-efficiency ancient recipe for the treatment of acute and chronic urticaria, ichthyosis in children and other skin itching diseases, with significant curative effects [11, 12]. Therefore, this work intended to apply ephedra-forsythia-red bean decoction in the treatment of psoriasis vulgaris.

As a new technical discipline, skin imaging has been gradually paid attention to in the field of dermatology with its unique advantages, and is widely used in disease diagnosis, postoperative evaluation, and efficacy observation, with the advantages of nonseminal, safety, simplicity, and repeatability [13]. Laser scanning confocal microscopy is a common technique developed and widely used in the mid-1980s, which combines technologies such as laser, electronic camera, computer image processing, and traditional optical microscope, so it is more and more widely used in biology and medicine and become an essential tool for biomedical experimental research [14, 15]. At present, the application of laser scanning confocal microscope in psoriasis mainly involves the analysis of typical symptoms such as psoriasiform hyperplasia, dermal papillary telangiectasia, and Munro microabscess [16]. Studies showed that the thickness and echo of various skin layers and their appendages are different when 15 MHz or higher frequency ultrasound is used to measure a variety of skin diseases, and analysis of these data will have a high reference value for the diagnosis, efficacy observation, and prognosis evaluation of skin diseases [17, 18]. High-frequency ultrasound can detect the thickness of skin lesions before and after rehabilitation of psoriasis vulgaris, providing an intuitive, simple, and objective index [19]. Therefore, 22 MHz ultrasound was used to analyze the changes of psoriasis vulgaris before and after traditional Chinese medicine treatment and provide a reference for the clinical treatment of psoriasis in this work.

In summary, applying high-frequency ultrasound in the evaluation of clinical efficacy of psoriasis is currently an ideal method. Based on this, 80 cases of psoriasis vulgaris with exterior closing and internal depression syndrome were collected as the study subjects. They were rolled into the observation group using ephedra-forsythia-red bean decoction addition and subtraction and the control group (traditional Chinese medicine of observation group subtraction of raw ephedra, cinnamomum cassia, addition of fineleaf schizonepeta herb, divaricate saposhnikovia root) for 22 MHz high-frequency ultrasonography. It aims to investigate the efficacy of ephedra-forsythia-red bean decoction addition and subtraction based on 22 MHz high-frequency ultrasound in the treatment of psoriasis vulgaris by comparing the lesion area, lesion severity, psoriasis area and severity index (PASI) score, and efficacy indicators before and after treatment between the two groups.

## 2. Materials and Methods

### 2.1. Research Objects

Eighty cases of psoriasis vulgaris with exterior closing and internal depression syndrome diagnosed and treated in the hospital from December 2018 to October 2020 were collected as the study subjects. The patients were randomly divided into control group (*n* = 40) treated with oral Chinese medicine (ephedra-forsythia-red bean decoction addition and subtraction) and control group (*n* = 40) treated with oral Chinese medicine (traditional Chinese medicine of observation group subtraction of raw ephedra, cinnamomum cassia, addition of fineleaf schizonepeta herb, divaricate saposhnikovia root). The study had been approved by the ethics committee of hospital, the patients and their families understood the study and signed the informed consent form.

Inclusion criteria: (1) patients with good compliance; (2) Patients who agreed to sign the informed consent; (3) Patients over 18 years old; (4) Patients with plaque skin lesions in the past year; and (5) Patients who were not received drug treatment in the past year.

Exclusion criteria: (1) patients who are allergic to the ingredients of traditional Chinese medicine; (2) Women who are lactating; (3) Women who have a fertility plan within one year; (4) Patients with clinical stage of regression; (5) Patients with malignant tumors or mental diseases; (6) Patients with incomplete case data; and (7) Patients in a stress state such as infection and trauma.

### 2.2. Discontinuation of Cases

(1) In the process of the experiment, if the patient's condition suddenly worsens, more serious complications occur, and even endanger life, the experiment will be immediately stopped. (2) In the process of the experiment, if the patient's traditional Chinese medicine syndrome changes, the physician can no longer use the drug of this study for intervention, the experiment will be immediately stopped. (3) In the process of the experiment, the physician will decide whether to terminate the experiment according to the medical ethical situation. (4) In the experiment, if there are poor compliance, trauma, accidents, and other deviations, the experiment will be immediately terminated. (5) The patient believes that there is a lack of efficacy in the treatment process, and requests to withdraw from this experiment. (6) The patient is reluctant to continue this experiment during the treatment due to his/her own factors, which should be immediately terminated.

### 2.3. Treatment Methods

Patients in the experimental group were treated with below formula: 6 grams of raw ephedra, 12 grams of fermented soybean, 10 grams of red bean, 9 grams of bitter apricot seed, 10 grams of cassia twig, 10 grams of white mulberry root-bark, 9 grams of weeping forsythia capsule, 15 grams of unprocessed rehmannia root, 10 grams of baical skullcap root, 6 grams of liquorice root. Clinical addition and subtraction: (1) people with headache and stuffy nose can optionally add flower bud of lily magnolia, manchurian wildginger, dahurian angelica root, siberian cocklebur fruit; (2) people who eat poorly can optionally add fried millet sprout, medicated leaven, fried germinated barley; (3) patients with sore throat can choose to add figwort root, great burdock achene, platycodon root; (4) Constipated persons can optionally add mirabilite and raw rhubarb; (5) persons with thirst and polydipsia can optionally add reed rhizome, gypsum.

Patients in the control group were treated with below formula: 9 grams of weeping forsythia capsule, 12 grams of fermented soybean, 6 grams of bitter apricot seed, 10 grams of white mulberry root-bark, 10 grams of fineleaf schizonepeta herb, 10 grams of red bean, 10 grams of divaricate saposhnikovia root, 15 grams of unprocessed rehmannia root, 10 grams of baical skullcap root, 6 grams of liquorice root. Clinical addition and subtraction are as above.

The specific mode of administration is the same: patients were given oral medicine, 450 mL decoction, 1 dose per day, morning, and evening after meals once. Two weeks is a course of treatment.

### 2.4. High-Frequency Ultrasonography

The skin ultrasonic diagnostic apparatus was used for examination with a probe frequency of 22 MHz. Before the examination, the probe was patched and appropriate amount of water was added, so that the patient could select the appropriate examination position according to different damaged sites. Coupling agent was applied to the lesion site, the probe was placed above the skin, slowly closed to the skin, performed dynamic scanning (transverse or longitudinal) after starting. The obtained ultrasound images were delivered to the workstation for processing and analysis, the percentage of skin lesions in the face and neck (head, face, and neck), upper limbs, trunk, and lower limbs to the total body surface area was measured, and the lesion severity score and psoriasis area and severity index (PASI) were recorded.

All 10 nails of each patient were examined using high-frequency ultrasound and arthroscopy, and if symptoms such as thickening, depression, roughness, detachment, discoloration, and transverse ridges were found, it was positive. The detection rates of psoriatic nail lesions by ultrasound and arthroscopy were calculated, respectively.

### 2.5. Observation Indexes

General data: gender, age, occupation, sleep status, date of visit, smoking history, drinking history, height, weight, allergy history, education level, and marital status.

Incidence: time of first onset, course of disease, duration of each onset, seasonality, inducement, complications, treatment in the past month, whether there is long-term medication for other diseases, visual analogue scale, family history, etc.

Special data: pruritus score, patient's subjective symptoms, rash site, area, shape, affected site (fingernails, toenails, scalp, joints), and isomorphic reaction.

### 2.6. Statistical Methods

The data processing of this study was analyzed by SPSS 19.0 statistical software. The measurement data were expressed as mean ± standard deviation ($\bar{\text{x}}$ ± *s*), and the enumeration data were expressed as percentage (%). One-way analysis of variance was used for pairwise comparisons. The difference was statistically significant at *P* < 0.05.

## 3. Results

### 3.1. Comparison of General Data of Two Groups of Patients

**Figure 1:**
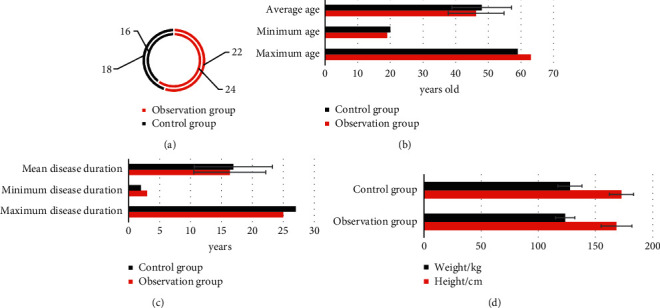
Comparison of general data of patients between two groups. (a) The number of males and females; (b) the maximum age, minimum age, and mean age; (c) the maximum disease course, minimum disease course, and mean disease course; (d) the height and weight.

Figure 1 shows there were no significant differences in the number of males and females, age (maximum age, minimum age, mean age), disease duration (maximum disease duration, minimum disease duration, mean disease duration), height, and weight between the observation group and the control group (*P* > 0.05).

### 3.2. Lesion Status before and after Treatment in Both Groups

**Figure 2:**
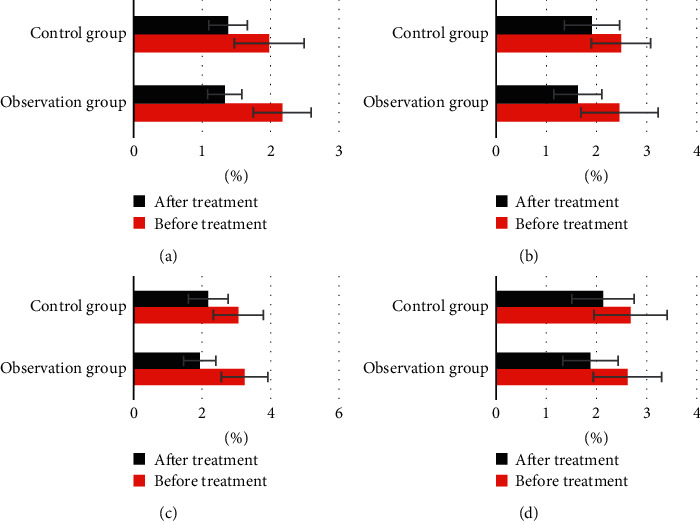
Patients' skin lesion area in observation group and control group before and after treatment. (a) Head, face, and neck; (b) upper limb; (c) trunk; (d) lower limb.

Figure 2 suggests there was no significant difference in the lesion area of head, face, neck, upper limbs, trunk, and lower limbs before treatment between the two groups (*P* > 0.05). The lesion area of head, face, neck, upper limbs, trunk, and lower limbs after treatment in the two groups was significantly lower than that before treatment, and the difference had statistical significance (*P* < 0.05). The lesion area of head, face, neck, upper limbs, trunk, and lower limbs after treatment in the observation group was lower than that in the control group, but there was no significant difference in the pairwise comparison (*P* > 0.05).

**Figure 3:**
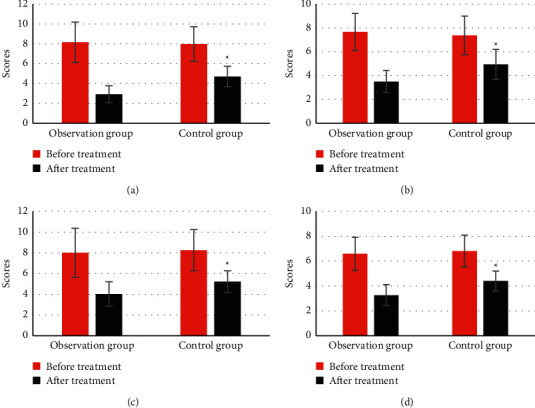
Severity of skin lesions in observation group and control group before and after treatment. (a) Head, face, and neck; (b) upper limb; (c) trunk; (d) lower limb. ^*∗*^ indicated that the difference between the observation group and the control group was statistically significant (*P* < 0.05).

Figure 3 reveals there was no significant difference in the severity of skin lesions on the head, face, neck, upper limbs, trunk, and lower limbs between the two groups before treatment (*P* > 0.05). The severity of skin lesions on the head, face, neck, upper limbs, trunk, and lower limbs in the two groups after treatment was significantly lower than that before treatment, and the difference had statistical significance (*P* < 0.05). The severity of skin lesions on the head, face, neck, upper limbs, trunk, and lower limbs in the observation group after treatment was lower than that in the control group, and the difference had statistical significance (*P* < 0.05).

### 3.3. Comparison of PASI Scores before and after Treatment between the Two Groups

**Figure 4:**
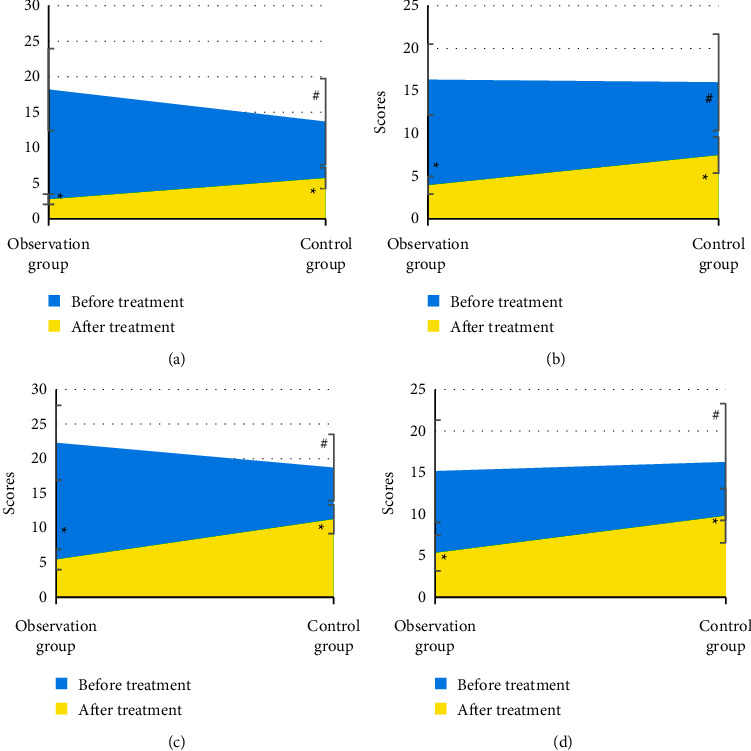
Comparison of PASI scores between the two groups before and after treatment. (a) Head, face, and neck; (b) upper limb; (c) trunk; (d) lower limb. ^*∗*^ showed difference between before and after treatment was statistically significant (*P* < 0.05); # indicated that the difference between the observation group and the control group was statistically significant (*P* < 0.05).

Figure 4 indicates there was no significant difference in PASI scores of head, face, neck, upper limbs, trunk, and lower limbs between the two groups before treatment (*P* > 0.05). The PASI scores of head, face, neck, upper limbs, trunk, and lower limbs in the two groups after treatment were significantly lower than those before treatment, and the difference had statistical significance (*P* < 0.05). The PASI scores of head, face, neck, upper limbs, trunk, and lower limbs in the observation group after treatment were lower than those in the control group, and the difference had statistical significance (*P* < 0.05).

**Figure 5:**
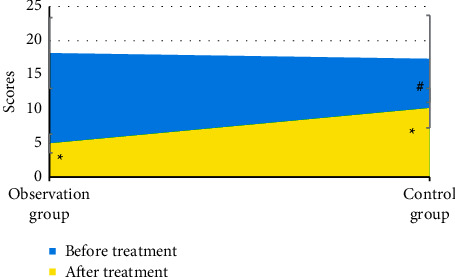
Comparison of total PASI score between the two groups before and after treatment. ^*∗*^ indicated that the difference between before and after treatment was statistically significant (*P* < 0.05); # indicated that the difference between the observation group and the control group was statistically significant (*P* < 0.05).

There was no significant difference in the total PASI score before treatment between the two groups (*P* > 0.05) (Figure 5). The total PASI score after treatment in the two groups was significantly lower than that before treatment, and the difference had statistical significance (*P* < 0.05), and that in the observation group was lower than that in the control group, and the difference had statistical significance (*P* < 0.05).

### 3.4. Comparison of Curative Effect Indexes between Observation Group and Control Group

**Figure 6:**
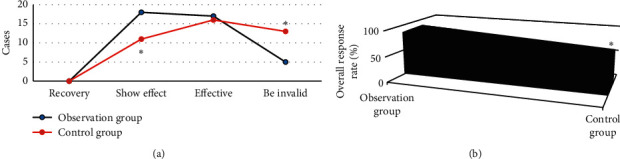
Comparison of efficacy indicators between the two groups after treatment. (a) The number of cured, markedly effective, effective, and ineffective cases; (b) the total efficiency. ^*∗*^Significant difference between the observation group and the control group (*P* < 0.05).

Figure 6(a) shows after treatment, 0 case was cured, 18 cases were markedly effective, 17 cases were effective, and 5 cases were ineffective in the observation group; after treatment, 0 case was cured, 11 cases were markedly effective, 16 cases were effective, and 13 cases were ineffective in the control group. Among them, the number of significantly effective cases in the observation group was significantly more than that in the control group, and the difference had statistical significance (*P* < 0.05). The number of ineffective cases in the observation group was significantly less than that in the control group, and the difference had statistical significance (*P* < 0.05).

Figure 6(b) shows the overall response rate of the observation group (87.5%) was significantly higher than that of the control group (67.5%), and the difference had statistical significance (*P* < 0.05).

### 3.5. Detection Results of Psoriatic Nail Lesions by High-Frequency Ultrasound and Arthroscopy

**Figure 7:**
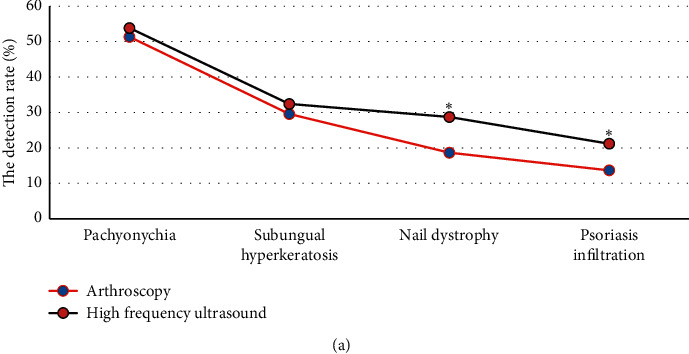
Detection results of psoriasis nail lesions by high-frequency ultrasound and arthroscopy. ^*∗*^Difference between high-frequency ultrasound and arthroscopy was statistically significant (*P* < 0.05).

The detection rate of nail plate thickening by arthroscopy was 51.35%, the detection rate of excessive nail angle was 29.61%, the detection rate of nail malnutrition was 18.63%, and the detection rate of psoriasis infiltration was 13.66% (Figure 7). The detection rate of nail plate thickening by high-frequency ultrasound was 53.85%, the detection rate of excessive nail angle was 32.4%, the detection rate of nail malnutrition was 28.74%, and the detection rate of psoriasis infiltration was 21.18%. There was no significant difference in the detection rate of nail plate thickening and excessive nail angle between high-frequency ultrasound and arthroscopy (*P* > 0.05). The detection rate of nail dystrophy and psoriatic infiltration in psoriatic nail lesions by high-frequency ultrasound was significantly higher than that by arthroscopy, and the difference was significant (*P* < 0.05).

### 3.6. Psoriasis Ultrasound Image Characteristics

**Figure 8:**
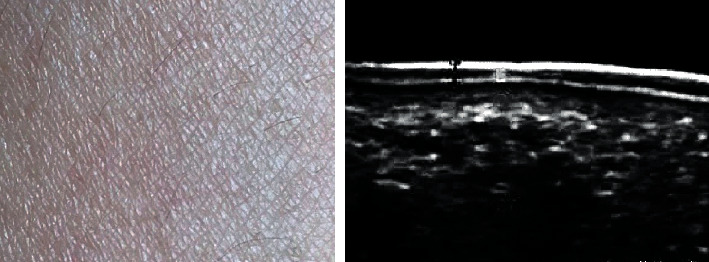
Normal skin and ultrasound images.

Figure 8 shows normal skin and its ultrasound images. There are two strong echo bands, with a wide medium echo band in the middle. The first layer of strong echo band is caused by the difference in acoustic impedance between the surface layer and the coupler interface; the echo of the second dermal layer is lower than that of the first layer, showing dense, medium-superimposed echo or short-fine echo, which is related to the closely arranged fibrous tissue in the dermis. The echo of the third layer of the subcutaneous layer varies from no echo to low echo, which contains a strong echo cord formed by dividing the fat lobular fibrous tissue.

**Figure 9:**
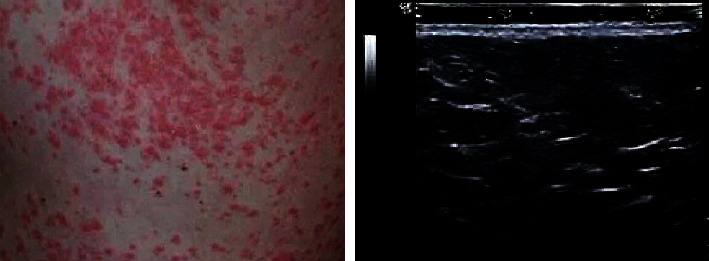
Psoriasis skin and ultrasound images.

Figure 9 is a case of psoriasis skin and its ultrasound images. Skin thickening, *epidermis* thickening, echo enhancement, dermal echo reduction, and no echo or very low echo dark band can be observed, and stationary phase subsided.

## 4. Discussion

Psoriasis, as a common dermatological disease, is characterized by protracted, recurrent, and incompletely eradicated. Although it generally does not endanger life, it causes great pressure on patients economically, physically, and psychologically due to the characteristics of the disease itself [20]. At present, there are many treatment methods for psoriasis, including topical western medicine, such as calcipotriol, glucocorticoids, and tazarotene. Traditional Chinese medicine treatment can play an important role based on traditional treatment, especially for psoriasis that is refractory to traditional treatment or easily recurs after drug withdrawal. The application of traditional Chinese medicine therapy often has unexpected efficacy, and it has advantages over western medicine treatment in terms of side effects and economic costs [21, 22]. Therefore, 80 cases of psoriasis vulgaris with exterior closing and internal depression syndrome diagnosed and treated in the hospital were selected as the study subjects and divided into 40 cases of observation group and 40 cases of control group. The observation group used oral Chinese medicine, and the control group used oral Chinese medicine, all of which underwent ultrasonic skin scanning [23]. Firstly, the basic data of the two groups were analyzed. It was found that there was no significant difference in the number of males and females, age (maximum age, minimum age, mean age), disease course (maximum disease course, minimum disease course, mean disease course), height, and weight between the observation group and the control group (*P* > 0.05), which provided the feasibility for subsequent study. Analysis of the ultrasonographic features of psoriasis showed that the psoriatic skin was thickened, the epidermal layer was thickened, the echo was enhanced, the dermal echo was reduced, and anechoic or very hypoechoic dark band could be observed under ultrasound. It was significantly different from the ultrasonographic features of normal skin, indicating that high-frequency ultrasound could effectively evaluate the condition and prognosis of psoriasis patients [24].

The lesion areas of various sites before and after treatment in the two groups were compared, and it was found that the lesion areas of head, face, neck, upper limbs, trunk, and lower limbs after treatment in the two groups were significantly lower than those before treatment, and the difference was statistically significant (*P* < 0.05). Such results indicated that both traditional Chinese medicine treatment methods could improve psoriasis vulgaris. The lesion area of head, face, neck, upper limbs, trunk, and lower limbs in the observation group was lower than that in the control group after treatment, but there was no significant difference between the two groups (*P* > 0.05), which may be due to the short observation time, and the difference in the lesion area between the two methods was not significant [25, 26]. In terms of lesion severity score, the lesion severity of head, face, neck, upper limbs, trunk, and lower limbs in the observation group was lower than that in the control group after treatment, and the difference was statistically significant (*P* < 0.05). It meant that ephedra-forsythia-red bean decoction addition and subtraction had a better effect in the treatment of psoriasis vulgaris. Analysis of total PASI revealed that the total PASI scores of the two groups after treatment were significantly lower than those before treatment, and the total PASI score of the observation group after treatment were lower than those of the control group, and the difference had statistical significance (*P* < 0.05). Such results were similar to the study by Lackner et al. [27], indicating that the improvement effect of ephedra-forsythia-red bean decoction addition and subtraction in the treatment of psoriasis vulgaris was better than that of the control group. Further comparison of the efficacy indicators showed that the overall response rate (87.5%) in the observation group was significantly higher than that in the control group (67.5%), and the difference had statistical significance (*P* < 0.05). The results here were consistent with the above PASI score results, comprehensively indicating that ephedra-forsythia-red bean decoction addition and subtraction is an effective formula for the treatment of psoriasis vulgaris vulgaris with exterior closing and internal depression syndrome.

## 5. Conclusion

In this study, 80 cases of psoriasis vulgaris with exterior closing and internal depression syndrome diagnosed and treated were selected as the study subjects and divided into the observation group and the control group according to different oral Chinese medicine regimens, and 22 MHz high-frequency ultrasonography was also performed. It was found that high-frequency ultrasound could effectively show the condition and prognosis of psoriasis patients, and ephedra-forsythia-red bean decoction addition and subtraction was an effective Chinese herbal formula for the treatment of psoriasis vulgaris vulgaris with exterior closing and internal depression syndrome. However, the sample size of patients selected in this study was small and the source was single, there was no long-term follow-up observation, and there was a lack of long-term prognosis data of patients. These problems needed to be improved. In the next study, more patient samples were included, long-term follow-up and high-frequency ultrasound examination are performed for patients after treatment, and the selection effect of traditional Chinese medicine formula for psoriasis vulgaris was deeply explored. In conclusion, the results of this work provided a reference for the selection of traditional Chinese medicine formulas for psoriasis vulgaris and the clinical application of high-frequency ultrasound.

## Data Availability

The data used to support the findings of this study are available from the corresponding author upon request.

## References

[1] Carrascosa J. M., Bonanad C., Dauden E., Botella R. (2017). Martín A; en nombre del Grupo de Trabajo en Inflamación Sistémica en Psoriasis. Psoriasis and Nonalcoholic Fatty Liver Disease. *Actas Dermosifiliogr*.

[2] Chesaut R. M., DikeaS T.-N. (2018). A method of-managing severe traumatic brain injury in the absence-of intracranial-pressure monitoring: the imaging and clinical examination protocol. *Journal of Neurotrauma*.

[3] Xia J., Xie S. Y., Liu K. Q. (2020). Systemic evaluation of the relationship between psoriasis, psoriatic arthritis and osteoporosis: observational and Mendelian randomisation study. *Annals of the Rheumatic Diseases*.

[4] Hu M, Zhong Y, Xie S, Lv H, Lv Z (2021). Fuzzy system based medical image processing for brain disease prediction. *Frontiers in Neuroscience*.

[5] Zheng W. Z, Wu Y. J, Ma Z. J, Mai Y. P (2020). Is overparenting harmful to creativity?. *Creativity and Innovation Management*.

[6] Lee A, Fischer G (2018). Diagnosis and treatment of vulvar lichen sclerosus: an update for dermatologists. *American Journal of Clinical Dermatology*.

[7] Choi H, Uceda D. E, Dey A. K (2020). Treatment of psoriasis with biologic therapy is associated with improvement of coronary artery plaque lipid-rich necrotic core: results from a prospective, observational study. *Circ Cardiovasc Imaging*.

[8] Di Matteo A, Filippucci E, Cipolletta E (2018). Entheseal involvement in patients with systemic lupus erythematosus: an ultrasound study. *Rheumatology*.

[9] González-Cantero A, Gonzalez-Cantero J, Sanchez-Moya A. I (2019). Femoral artery ultrasound for improving the detection of atherosclerosis in psoriasis. *Journal of the American Academy of Dermatology*.

[10] Wang D, Lu C, Yu J, Zhang M, Zhu W, Gu J (2020). Chinese medicine for psoriasis vulgaris based on syndrome pattern: a network pharmacological study. *Evid Based Complement Alternat Med*.

[11] Chen X, Zhang R, Duan X, Xue M, Qu T, Li L (2020). Effectiveness of Xiaoyin Jiedu granules in the treatment of psoriasis vulgaris in patients with blood-heat symptom patterns in terms of Traditional Chinese Medicine. *Journal of Traditional Chinese Medicine*.

[12] Deng J, Yao D, Lu C (2017). Oral Chinese herbal medicine for psoriasis vulgaris: protocol for a randomised, double-blind, double-dummy, multicentre clinical trial. *BMJ Open*.

[13] Gelfand J. M, Shin D. B, Duffin K. C (2020). A randomized placebo-controlled trial of secukinumab on aortic vascular inflammation in moderate-to-severe psoriasis vulgaris (VIP-S). *Journal of Investigative Dermatology*.

[14] Gonzalez-Cantero A, Gonzalez-Cantero J, Sanchez-Moya A. I (2019). Subclinical atherosclerosis in psoriasis. Usefulness of femoral artery ultrasound for the diagnosis, and analysis of its relationship with insulin resistance. *PLoS One*.

[15] Krajewska-Włodarczyk M, Owczarczyk-Saczonek A, Placek W, Wojtkiewicz M, Wiktorowicz A, Wojtkiewicz J (2018). Ultrasound assessment of changes in nails in psoriasis and psoriatic arthritis. *BioMed Research International*.

[16] Solmaz D, Bakirci S, Al Onazi A, Al Osaimi N, Fahim S, Aydin S. Z (2020). Musculoskeletal ultrasound can improve referrals from dermatology to rheumatology for patients with psoriasis. *British Journal of Dermatology*.

[17] Elnabawi Y. A, Dey A. K, Goyal A (2019). Coronary artery plaque characteristics and treatment with biologic therapy in severe psoriasis: results from a prospective observational study. *Cardiovascular Research*.

[18] Naredo E, Janta I, Baniandrés-Rodríguez O (2019). To what extend is nail ultrasound discriminative between psoriasis, psoriatic arthritis and healthy subjects?. *Rheumatology International*.

[19] Elnabawi Y. A, Oikonomou E. K, Dey A. K (2019). Association of biologic therapy with coronary inflammation in patients with psoriasis as assessed by perivascular fat attenuation index. *JAMA Cardiol*.

[20] Lifshiz Zimon R, Lerman G, Elharrar E (2018). Ultrasound targeting of Q-starch/miR-197 complexes for topical treatment of psoriasis. *Journal of Controlled Release*.

[21] Wan Z, Dong Y, Yu Z, Lv H, Lv Z (2021). Semi-supervised support vector machine for digital twins based brain image fusion. *Frontiers in Neuroscience*.

[22] Solmaz D, Bakirci S, Jibri Z, Sampaio M, Karsh J, Aydin S. Z (2020). Psoriasis is an independent risk factor for entheseal damage in axial spondyloarthritis. *Seminars in Arthritis and Rheumatism*.

[23] Micali G, Verzì A. E, Musumeci M. L, Luca M, Lacarrubba F (2019). Ultrasound assessment of the keratolytic effect of a 50% urea anhydrous paste on psoriasis plaques: a prospective study. *Giornale Italiano di Dermatologia e Venereologia*.

[24] Ortolan A, Lorenzin M, Tadiotto G (2019). Metabolic syndrome, non-alcoholic fatty liver disease and liver stiffness in psoriatic arthritis and psoriasis patients. *Clinical Rheumatology*.

[25] Kamath P, Benesh G, Romanelli P, Iacobellis G (2019). Epicardial fat: a new therapeutic target in psoriasis. *Current Pharmaceutical Design*.

[26] Khandpur S, Yadav D, Jangid B (2020). Ultrasound liver elastography for the detection of liver fibrosis in patients with psoriasis and reactive arthritis on long-term methotrexate therapy: a cross-sectional study. *Indian Journal of Dermatology Venereology and Leprology*.

[27] Lackner A, Heber D, Bosch P (2020). Ultrasound verified enthesophytes are associated with radiographic progression at entheses in psoriatic arthritis. *Rheumatology*.

